# Chicken‐or‐egg question: Which came first, extracellular vesicles or autoimmune diseases?

**DOI:** 10.1002/JLB.3MR0120-232R

**Published:** 2020-02-28

**Authors:** Federica Maione, Giuseppe Cappellano, Mattia Bellan, Davide Raineri, Annalisa Chiocchetti

**Affiliations:** ^1^ Center for Translational Research on Autoimmune and Allergic Disease‐CAAD Università del Piemonte Orientale Novara Italy; ^2^ Department of Health Sciences Interdisciplinary Research Center of Autoimmune Diseases‐ IRCAD Università del Piemonte Orientale Novara Italy; ^3^ Department of Translational Medicine Università del Piemonte Orientale Novara Italy

**Keywords:** autoimmune diseases, diagnosis, extracellular vesicles, flow cytometry, pathogenesis, therapeutics

## Abstract

Extracellular vesicles (EVs) have attracted great interest as contributors to autoimmune disease (AD) pathogenesis, owing to their immunomodulatory potential; they may also play a role in triggering tolerance disruption, by delivering auto‐antigens. EVs are released by almost all cell types, and afford paracrine or distal cell communication, functioning as biological carriers of active molecules including lipids, proteins, and nucleic acids. Depending on stimuli from the external microenvironment or on their cargo, EVs can promote or suppress immune responses. ADs are triggered by inappropriate immune‐system activation against the self, but their precise etiology is still poorly understood. Accumulating evidence indicates that lifestyle and diet have a strong impact on their clinical onset and development. However, to date the mechanisms underlying AD pathogenesis are not fully clarified, and reliable markers, which would provide early prediction and disease progression monitoring, are lacking. In this connection, EVs have recently been indicated as a promising source of AD biomarkers. Although EV isolation is currently based on differential centrifugation or density‐gradient ultracentrifugation, the resulting co‐isolation of contaminants (i.e., protein aggregates), and the pooling of all EVs in one sample, limit this approach to abundantly‐expressed EVs. Flow cytometry is one of the most promising methods for detecting EVs as biomarkers, and may have diagnostic applications. Furthermore, very recent findings describe a new method for identifying and sorting EVs by flow cytometry from freshly collected body fluids, based on specific EV surface markers.

## Graphical abstract

Review on the role of EVs in autoimmune diseases, highlighting their importance as novel biomarkers and their potential role in triggering loss of tolerance against self‐antigens.

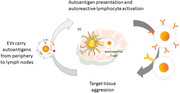

**Abbreviation**
ACPA: anti‐citrullinated protein
AD: autoimmune diseases
ANAs: anti‐nuclear antibodies
AnxA1: annexin A
AQPs: aquaporins
BBB: blood–brain barrier
bDMARDs: disease
BMEVs: bovine‐milk‐derived EVs
CD2AP: CD2 Associated Protein
CFS: cerebrospinal fluid
CIA: collagen‐induced arthritis
CIS: clinically isolated syndrome
COX‐2: cyclooxygenase 2
CRP: reactive C protein
DAS: disease activity score
DC: dendritic cell
EAE: experimental autoimmune encephalomyelitis
ECM: extracellular matrix
ERV: endogenous retrovirus
EV: extracellular vesicle
FLS: fibroblast‐like synoviocyte
GAD65: glutamic acid decarboxylase 65
HC: healthy control
IA‐2: islet autoantibodies‐2
ICs: immune complexes
LCAP: leukocytapheresis
LN: lupus glomerulonephritis
MLV: murine leukemia retrovirus
MMPs: matrix metalloproteases
mPGES‐1: microsomal prostaglandin E synthase 1
MS: multiple sclerosis
MSC: mesenchymal stem cell
MVB: multivesicular bodie
MV: microvesicle
OIND: other inflammatory neurological disorder
PAD: peptidylarginine deiminase
PMP: platelet‐derived microparticles
RA: rheumatoid arthritis
RM: Rheumatoid Factor
RR: relapsing‐remitting
SAP: serum amyloid‐P
SF: synovial fluid
SLE: systemic lupus erythematosus
T1D: type I diabetes
TMEV: Theiler's murine encephalomyelitis virus
Treg: regulatory T cell
tsDMARDs: targeted synthetic disease modifying antirheumatic drugs

## 1 INTRODUCTION

This review will describe the role of extracellular vesicles (EVs) in the pathogenesis of four autoimmune diseases, namely type 1 diabetes (T1D), multiple sclerosis (MS), rheumatoid arthritis (RA), and systemic lupus erythematosus (SLE), also examining the possibility of applying them as diagnostic or therapeutic‐response biomarkers, and their great potential as therapeutics.

### 1.1 Role and characteristics of EVs

EVs are lipid‐bound vesicles released in biological fluids (e.g., blood, urine, breast milk, saliva, and cerebrospinal fluids or amniotic fluids) and in solid tissues, by almost all cell types, and increased in response to various stimuli (i.e., cell activation, apoptosis, and mechanical injury). They represent an alternative mechanism for cell‐to‐cell communication and are required either to maintain tissue homeostasis and to activate a response to pathogens in the extracellular space. Although their presence within human peripheral blood had long been known, EVs were first described in 1996 as a way to exchange information between different cells, indicating their possible involvement in antigen presentation.^1^, ^2^

Studies focused on EVs have recently intensified, in order to better characterize cell‐derived vesicles. EVs have been classified by size and cell‐type origin; depending on their size, there are four main types of EV: (1) microvesicles (MVs) (100–1000 nm in diameter); (2) apoptotic blebs (1000–5000 nm in diameter); exosomes (20–150 nm), and multivesicular bodies (MVBs).^3^ Basically, MVs and apoptotic blebs originate by outward protrusion of the plasma membrane, whereas exosomes and MVBs are generated by invagination of the plasma membrane.^4^

### 1.2 Biological relevance of EVs

EVs are thought to function as shuttles for active molecules, such as proteins, lipids, metabolites, nucleic acids, in the exchange between different cells within an organism.^5^ Depending on the nature of the body fluid and on the cell type, once EVs have interacted with target cells they can participate in immune‐modulation, induce angiogenesis, promote coagulation, initiate apoptosis based on the active components carried by particles, or may control neuronal development, cellular proliferation, differentiation, and senescence (Table 1).

**TABLE 1:** Biological effects of active molecules carried by EVs

Molecule	Origin	Contents	Role
Lipids	Parental cell plasma membrane	Sphingosine‐1‐phosphate, phosphatidylserine (PS), cholesterol and arachidonic acid	Regulation of distal immune‐response, activation of pro‐coagulant cascade
Proteins	Parental cell plasma membrane and cytoplasm	proteins involved in the vesicles’ trafficking or endocytic pathways, cell surface receptors, cytokines, hormones, growth and transcription factors, and heat‐shock proteins	Regulation of immune response and inflammation, angiogenesis, coagulation, autophagy, apoptosis
Nucleic acids	Parental cell RNA/DNA	microRNA, mRNA, RNA, and DNA.	Regulation of gene expression and protein synthesis in target cells

### 1.3 EVs in regulation of immune response

EVs from both immune and non‐immune cells, such as mesenchymal stem cells (MSCs) and endothelial cells (ECs), contribute to antigen‐specific and aspecific immune regulation. EVs exert their immunoregulatory functions through paracrine mechanisms and can promote or suppress immune responses.^6^ In particular, APCs, including dendritic cells (DCs), macrophages, and B cells, regulate immune responses through direct interaction with CD4^+^ and CD8^+^ T cells and other immune cell types, such as NK and NKT cells. The activity of APCs on the immune function is mediated by several surface proteins (such as MHC class I and II costimulatory molecules and adhesion molecules), which are also present in EVs released by these cells. The current hypothesis is that the presence of immune regulatory proteins within the EVs enables APCs to modulate T cells at a distance. This regulation can be achieved through two different mechanisms: (i) a percentage of EVs remain attached to the APCs in an appropriate orientation in specific areas of their plasma membrane, so that the MHC complexes carried by the EVs are presented without further processing to cognate T cells (cross‐dressing); (ii) EVs can deliver native antigens to APCs by internalization and processing, and EV‐derived peptides can be used for presentation to T cells.^7^

### 1.4 EVs in the etiology of autoimmune diseases

**FIGURE 1:**
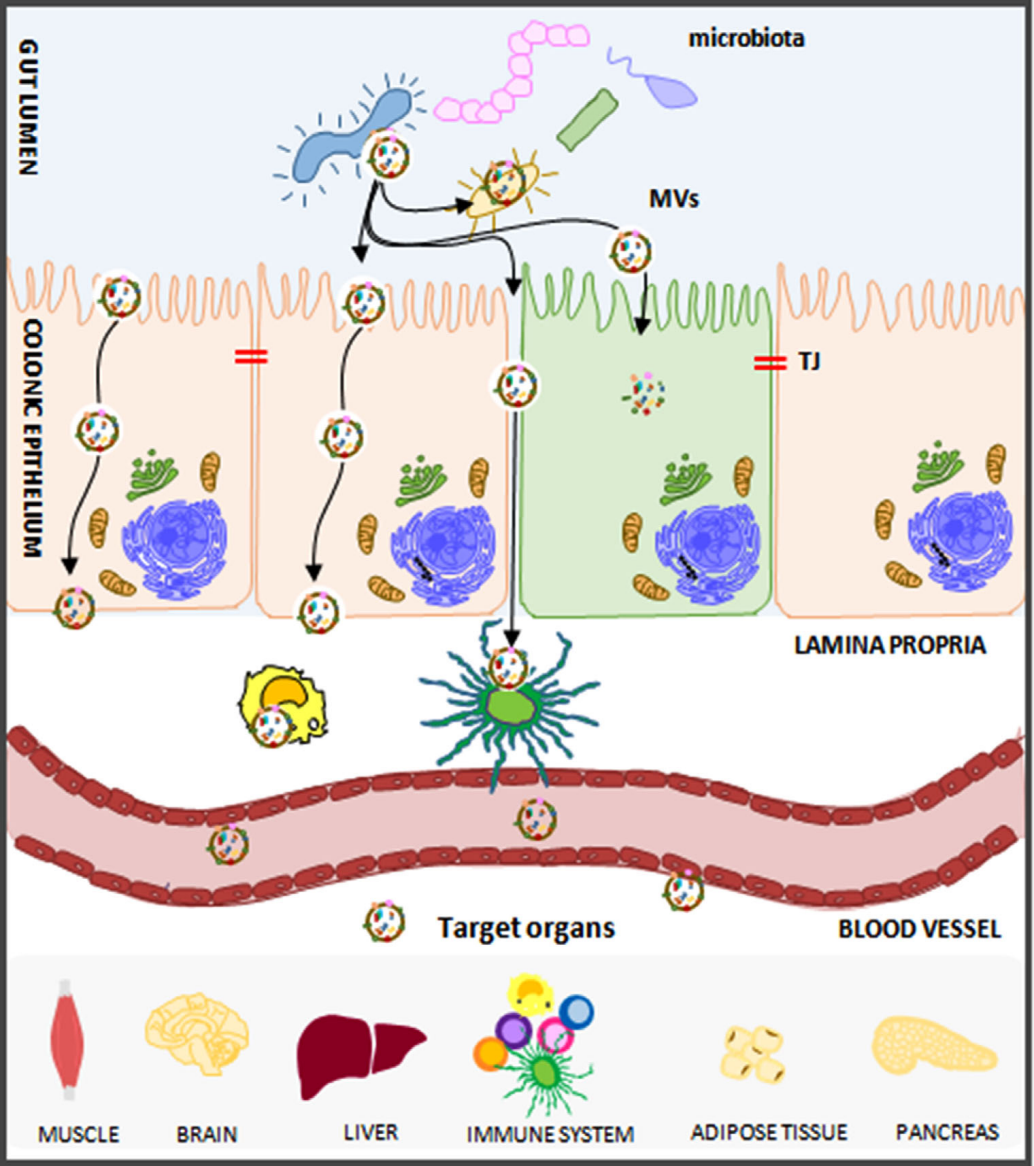
**Microbial communities secrete EVs that can deliver positive or negative messages to the body**. Not only damaged tissues, but also microbiota and food from the gut lumen, seem to deliver EVs or their content to epithelial cells and/or enter the systemic circulation and may be delivered to different proximal or distant organs, eliciting a variety of immunological and metabolic responses. Once that EVs or access and cross the gut epithelial barrier, they can interact with immune cells in the gut‐associated lymphoid tissue (GALT) and with the different targets by systemic circulation

Genetic factors clearly predispose to the development of autoimmune diseases (AD),^8^ but environmental factors are also important triggers. Epidemiologic studies strongly suggest that the rate of ADs is increasing over time, especially in industrialized countries, depending on lifestyle. In particular, nutritional patterns collectively termed the “Western diet” (WD; high‐fat/cholesterol, high‐protein, high‐sugar, and excess salt intake) together with the frequent consumption of processed and “fast foods” have attracted interest as possible promoters of AD. Diet and the consumption of highly processed foods impact on the intestinal epithelium, which is the primary absorption interface for nutrients. In normal conditions, the intestinal epithelium functions as a barrier between host and environment, including food and microbiota. The term "microbiota" indicates the community of commensal, symbiotic, or pathogenic microorganisms that reside within the human body. In pathologic conditions, this barrier may be altered, creating a “leaky gut” that allows the passage of toxins, food antigens, as well as bacteria, into the bloodstream. In individuals with a genetic predisposition, a leaky gut may trigger the initiation and development of AD. The association between dysbiosis and AD has been characterized in‐depth, but the impact of precise communities or species of microbe is not yet fully characterized, and further studies are required. However, it is interesting to note that the microbiota is shaped in the first years of life (native core microbiota) and that, once formed, it is resistant and resilient against perturbations.^9^ It is noteworthy that organisms of all three kingdoms (including plants and bacteria) produce EVs. It has thus been suggested that microbial communities may secrete EVs and deliver positive or negative messages to the body (Fig. 1).

The role of EVs in AD‐associated chronic inflammation reportedly varies, leading to tissue damage and premature aging. Interestingly, EV secretion and activity appears to change with physiological and pathological aging: for instance, mesenchymal stem cells (MSCs) from old rats secrete greater quantities of EVs than do those of their young counterparts; the EV composition also differs, since ‘elderly’ EVs have a lower content of anti‐inflammatory miRNAs, and in particular of miR‐133b‐3p and miR‐294, which inhibit the transforming growth factor (TGF−β)−mediated epithelial‐to‐mesenchymal transition. This latter process contributes to renal fibrosis, a complication often occurring in AD patients. Moreover, in these patients, the aging of the immune system, which entails the decline of immune protective adeptness and proinflammatory effector cell enrichment, may be accelerated. Therefore, to investigate the role of EVs in immune‐cell aging and inflammation is crucial for the development of novel interventions counteracting the detrimental effects of organismal aging and inflammatory disease.^10^, ^11^, ^12^

Conversely, EVs may also serve as therapeutic tools for ADs: they have low immunogenicity, long half‐life in circulation and, most importantly for MS and other neurodegenerative diseases, they can cross the blood‐brain barrier (BBB) even without any surface modification. Thus, EVs may also be used to deliver drugs directly into the targeted organ.

The possible involvement of EVs in the treatment of autoimmune disorders is based on two different characteristics of these vesicles. First, EVs are natural carriers of functional DNA, RNA, and proteins, making them a suitable tool to deliver macromolecules and/or synthetic drugs.^13^ Second, EVs can be modified on their surface markers in order to target specific tissues enhancing their therapeutic potential.^14^

## 2 TYPE 1 DIABETES

T1D is a metabolic disorder characterized by elevated blood glucose levels attributed to insufficient or absent production of insulin, as a consequence of autoimmune aggression leading to β‐pancreatic cell loss. Long‐term complications of the disease have been associated with macro‐ and micro‐vascular problems, leading to heart diseases, stroke, blindness, and kidney disease.^15^ Early diagnosis of diabetes mellitus could thus significantly improve preventive and therapeutic strategies. In this connection, there is a pressing need to identify biomarkers that could enable screening the population for the risk of developing diabetes, as well as affording a monitoring strategy for disease progression.

### 2.1 EVs in T1D pathogenesis

The endocrine pancreas comprises large clusters of cells known as islets of Langerhans, each containing thousands of cells. EVs contribute to the paracrine interactions among these cells, orchestrating hormonal secretion and promoting islet health and survival.^16^

**FIGURE 2:**
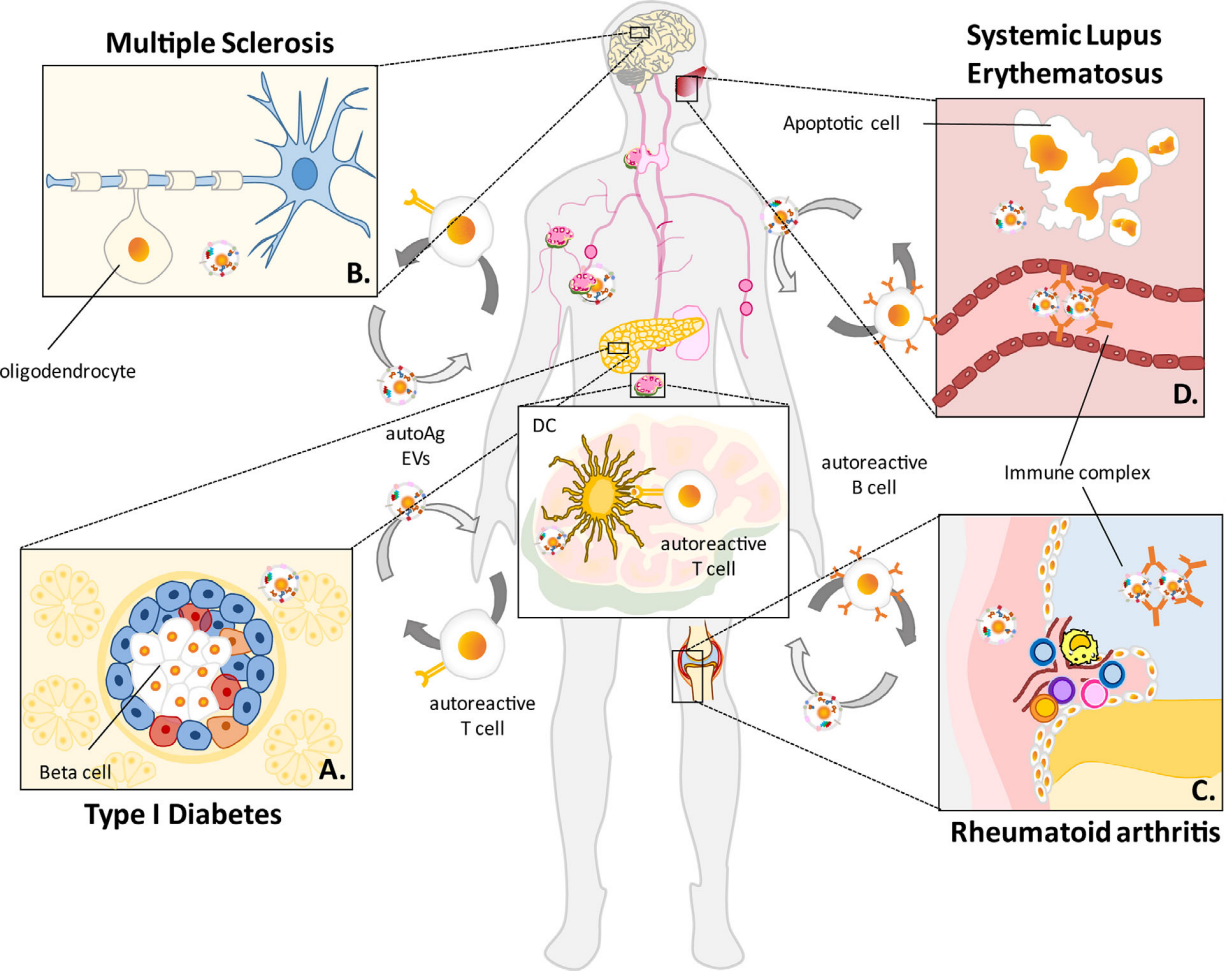
**EVs may trigger AD by delivering autoantigens to secondary lymphatic organs**. In ADs EVs can carry autoantigens (AutoAgs) to lymph nodes and deliver them to APCs. In T1D these AutoAgs are released by pancreatic β−cell (**A**), by oligodendrocytes in MS (**B**). In RA (**C**) and in SLE (**D**), EVs derived from synovial cells or apoptotic cells respectively, carry autoantigens that are recognized by autoreactive B cell, resulting in the formation of immunocomplexes

In the last decade, many studies have sought to characterize the nature of EVs cargo. Interestingly, in‐depth analysis of their content has revealed the presence of insulin, C‐peptide proteins, glutamic acid decarboxylase 65 (GAD65), low levels of glucagon and endothelial nitric oxide synthase, suggesting that they mostly originate from insulin‐producing ß‐cells.^17^ Further, many miRNAs have been found to be enriched in EVs derived from ß‐cells.^18^ Regarding T1D pathogenesis, the current hypothesis is that pro‐inflammatory cytokines secreted by immune cells within the pancreas contribute to creating an inflammatory microenvironment that, in turn, promotes the ß‐cells attack by the immune system.^19^ The exposure of these cells to inflammation promotes a primary islet inflammatory signaling, which triggers the loss of self‐tolerance leading to disease onset. Emerging evidence suggests that EVs play a crucial role in the initiation of autoimmune responses in the islets.^20^ For instance, ß‐cells exposed to pro‐inflammatory cytokines display an altered miRNA signature compared to unstimulated cells. Further, these EVs show an apoptotic‐inducing effect on recipient cells; they can also release intracellular ß‐cell autoantigens (i.e. GAD65, IA‐2, and proinsulin) which can be internalized by APCs resulting in autoreactive T and B cells activation.^21^, ^22^ (Fig. 2A)

Over 30 years ago, Leiter et al. discovered the presence of retroviral elements within EVs from T1D patients, and hypothesized that they could act as “mimetic antigens” to sensitize ß‐cells and trigger autoimmunity.^23^ Indeed, the presence of endogenous retroviruses (ERV), which are the germline‐integrated remnants of ancient retrovirus infections, is detectable in pancreatic islets of T1D patients.^23^ Using several monoclonal antibodies (mAbs), Tsumura et al. demonstrated that murine leukemia retrovirus (MLV) Gag and Env antigen expression is increased in non‐obese diabetic (NOD) mice islets.^24^ Recently, mAbs specific for MLV Env have been isolated from young NOD mice, and xenotropic ERV can be isolated from pancreatic β cell lines. Taken together, these observations point to a new role for EVs in T1D as carriers of ERV particles, suggesting the possibility that autoimmunity may be triggered as a byproduct of an immune response against retrovirus‐like MVs.^25^

### 2.2 EVs as T1D biomarkers

To date, biomarkers for predicting T1D risk are susceptibility genes (especially HLA genes involved in antigen presentation) and islet autoantibodies (i.e., GAD65, IA‐2, and pro‐insulin).^26^ However, these markers display several limitations: autoantibodies become detectable relatively late in the disease process, thus limiting their value in early disease prediction; further, IA‐2 autoantibodies tend to decrease with disease duration, and insulin autoantibodies cannot be used after starting insulin therapy.^27^ The identification of novel biomarkers is thus still an issue for the identification of new therapeutic targets and therapy improvement. EVs have recently been indicated as promising biomarkers. However, the active molecules present in EVs in T1D must also be understood in greater depth. It is known that the EV cargo contains miRNAs or proteins, including autoantibodies, that might be used for T1D diagnosis. Notably, islets from T1D patients have higher EV levels than healthy subjects.^25^ Several EV active molecules have been indicated as T1D‐inducing factors. For instance, Lakhter *et al* demonstrated that the serum increase of miR‐21‐5p in EV cargo could induce diabetes development.^28^

The clinical progression of diabetes is often associated with complications such as nephropathy, retinopathy, or microangiopathy. Notably, these complications may be related to aging of the T cell system.^11^ For instance, EVs originating from platelets and detected using lactadherin as vesicle marker have been found increased in T1D patients plasma with microangiopathy.^29^ Increased expression of proteases in EVs (i.e., cystatin B, prostasin, and urokinase) have been found in the urine and plasma and correlated with nephropathy and retinopathy.^30^ Conversely, multiple analyses of the miRNA expressed by EVs isolated from T1D patients serum/plasma indicated miR‐150‐5p, miR‐21‐3p, miR126, miR‐145, and miR‐30b‐5p as potential biomarkers of the onset of diabetic retinopath.^31^ Lastly, both an increased expression of water‐channel aquaporins (AQPs), and in particular of AQP5 and AQP2, in EVs derived from epithelial tubular cells, and higher amounts of podocyte‐derived EVs in the urine of T1D patients are associated with nephropathy development.^32^ Despite EV's potential in the context of T1D, preclinical and clinical studies are only now gaining importance. EV may be used as biomarker for the detection of early islet injury, and may serve to identify susceptible individuals for disease progression, before autoantibodies are detectable.

### 2.3 EVs for T1D therapy

Studies on NOD mice, an excellent model for studying genetic susceptibility to human T1D, show that both islet cells and MSCs exert an immunomodulatory effect by releasing highly‐immune‐stimulatory EVs.^33^ In particular, EVs derived from murine MSCs have been found to elicit a response in autoreactive T‐cells and marginal zone‐like B‐cells.^33^, ^34^ Of note, studies to evaluate the EV‐induced immune response demonstrated that splenic B cells from prediabetic NOD mice, but not from diabetic‐resistant mice (i.e. C57/Bl6 mice) responded to stimulation by exhibiting increased reactivity to EVs, thus indicating that EVs can activate APCs in NOD mice also in the absence of T cells.^35^ Stem cells are currently the main candidates for the development of new treatments for diabetic nephropathy, which is one of the most serious complications in diabetes patients. Preclinical observations demonstrated that, when injected into diabetic rats, EVs from urine‐derived stem cells, carrying transforming growth factor‐ß1, angiogenin, and bone morphogenetic protein‐7, can reduce the urine volume and urinary microalbumin excretion, prevent podocyte and tubular epithelial cell apoptosis, suppress caspase‐3 overexpression, and increase glomerular endothelial cell proliferation.^36^ Moreover, another study showed that EVs isolated from human islets stimulate proinflammatory immune responses, and lead to peripheral blood mononuclear cell (PBMC) activation.^17^ Additionally, they induce an increase in antibodies against GAD65 in PBMCs isolated from T1D patients. Furthermore, pretreatment of T1D derived PBMCs with ibrutinib, an inhibitor of Bruton tyrosine kinase that plays a crucial role in B cell maturation as well as in mast cell activation through the high‐affinity IgE receptor, dampens EV‐induced memory B cell activation and GAD65 antibody production.^37^

Conversely, as mentioned above, EVs could be employed as therapeutic tools in the delivery of specific miRNAs. An example of this approach is the fact that the transfer of miR145 by EVs derived from bone marrow stromal cells in diabetic rats conferred neuro‐restorative effects in a rat stroke model.^38^ In addition to nucleic acids, EVs have been successfully used to deliver drugs such as curcumin, which is a natural polyphenol with anti‐inflammatory properties, that had an effect on T1D mice after stroke, ameliorating neurovascular dysfunction.^39^

## 3 MULTIPLE SCLEROSIS

MS is an inflammatory demyelinating disease of the CNS caused by autoimmune aggression against myelin proteins.^40^ Among the different clinical patterns, relapsing‐remitting MS (RR‐MS) is the most common clinical form of the disease, with several periods of relapse, due to autoimmune aggression, and remission, due to immune system switching off.^41^, ^42^ MS, as well as other neurodegenerative disorders, is driven by inflammation that may exert either detrimental or protective functions, depending on local factors and on the timing of immune activation and shutting‐off systems.^43^

Diagnosis and MS monitoring rely on multiple clinical parameters, including clinical examination, magnetic resonance imaging, cerebrospinal fluid assessment, and electrophysiology, since currently no definitive biomarkers exist for diagnosis and prediction of MS evolution.

### 3.1 EVs in MS pathogenesis

The blood‐brain barrier (BBB) consists of a network of endothelial cells, together with neurons and glial cells, including microglia. Disruption of the BBB is considered an important feature contributing to MS pathogenesis.^44^ EVs may contribute to MS pathogenesis by spreading and amplifying CNS inflammation^45^ as documented by considerable experimental evidences. However, EVs may also exert a protective and regenerative effect in the repair of injury through different mechanisms including: restoration of trophic factors, control of synaptogenesis, or removal of damaged cells.^46^, ^47^ Endothelial EVs carry metalloproteases that may promote BBB disruption,^48^ molecules inducing endothelial activation,^49^ and sustain transendothelial migration of both monocytes and lymphocytes through the impaired BBB.^50^, ^51^ Plasma‐derived EVs from RR‐MS patients induced a stronger disruption of endothelial barriers than did those derived from healthy controls or patients with clinically isolated syndrome (CIS).^52^

Platelet‐derived EVs from MS patients increased the expression of integrins such as α4β1 (VLA‐4), promoting the binding of lymphocytes to the endothelium and favoring their migration across the BBB.^53^ Moreover, in MS, the endothelium/microglia crosstalk impacts on brain function.^54^ Microglia‐derived EVs are enriched in caspase 1, which has been shown to regulate the proteolytic activity of metalloproteases in endothelial cells.^54^, ^55^, ^56^ Moreover, the cargo of microglia‐derived‐EVs can also be transferred to neurons and, through its miRNAs, silence genes involved in dendritic spine formation and synaptic stability. In vivo, injection of EVs derived from inflammatory rat microglia, enriched in miR‐146a‐5p, resulted in the loss of dendritic spines in neurons.^57^ Furthermore, activated microglia EVs store and release interleukin (IL)‐1β^55^ and MHC‐II, propagating neuroinflammation, and providing an efficient route for rapid dissemination and epitope spreading.^58^

Regulatory T cells (Tregs) play an important role in CNS autoimmune inflammation, and their function is impaired in MS.^59^ Azimi et al. showed that Tregs‐derived exosomes from MS patients have a reduced capacity to suppress in vitro proliferation of effector T cells compared to those derived from healthy controls.^60^ Moreover, circulating plasma exosomes from MS patients suppress the induction of Treg cells on CD4^+^ naïve T cells, and this mechanism is miRNA‐mediated.^61^

The CNS is an immune‐privileged site, but since EVs can cross the BBB it may be speculated that EVs derived from brain cells might spread myelin antigens to the periphery, which would activate T cells before they enter the CNS^62^ (Fig. 2B **)**. In line with this possibility, Goetzl et al. reported that plasma astrocyte‐derived EVs in the circulation of patients with Alzheimer's disease were enriched with neuronal antigens.^63^

In summary, EVs may play a pathogenetic role in MS via several mechanisms, such as activation of T cells during relapses, supporting their migration through the BBB, and spreading inflammation within the CNS. Conversely, the diffusion of autoantigens in the periphery is still an open issue.

### 3.2 EVs as biomarkers for MS

CSF is the body fluid most closely reflecting the CNS in MS,^64^ but because of ethical concerns, the majority of studies focused on searching for biomarkers in the peripheral blood, which is more readily accessible than the CSF. Nevertheless, a recent study^65^ showed that tears contain microglia‐ and neural‐derived EVs, and proteomic analysis revealed that their content recapitulates that of CFS‐derived EVs, suggesting that tears might be a less invasive method for determining biomarkers in MS.

A pioneering study in MS showed that plasma CD31+ EVs were elevated during relapses compared to healthy control levels, returning to basal levels during remission phases.^49^ Marcos‐Ramiro et al. also reported elevated plasma EV levels in all clinical forms of MS, compared to both healthy controls and CIS patients.^52^ Interestingly, these EVs also had a synergetic effect with thrombin on the endothelial function. The study authors hypothesize that MS‐EVs may also potentiate the effects of proinflammatory mediators on endothelial barrier dysfunction, and suggest it would be interesting to characterize the cargo of these EVs to identify the molecules that are responsible for the observed effect.^52^

Verderio et al. described for the first time the presence of myeloid MVs in the CFS of MS patients and in healthy controls, but the MV concentration was increased in patients with RRMS or CIS, compared with both healthy controls and MS patients with stable disease. Interestingly the authors found a linear correlation between MVs concentration and gadolinium‐positive lesions at MRI, suggesting MVs as novel biomarkers to monitor MS progression.^66^ Furthering this research, a recent study characterized CFS‐EVs from MS patients, and confirmed the increase of EVs IB4^+^ (a marker of myeloid cells) in MS patients with relapse compared to those with stable disease. However, in this case, the concentration did not differ from that of MS patients with other inflammatory neurological disorder (OIND) or CIS.^67^ These opposing findings appear to suggest that the EV concentration is not a useful biomarker to differentiate MS patients from OIND or CIS.^67^ The differences between these two studies might, however, be ascribed to the different sample sizes.^66^, ^67^

MS therapy per se can affect the number of EVs. Plasma levels of B‐cell EVs were restored in MS patients treated with fingolimod, which is a second‐line therapeutic agent approved for the RR‐MS, while levels of endothelial EVs returned to the levels found in healthy controls. Conversely, the number of EVs from T cells or monocytes did not change upon treatment.^68^Collectively, these findings suggest the possibility of using EVs as biomarkers to monitor the response to therapy in MS. *In vitro*, fingolimod reduces the production of MVs derived from cultured monocytes purified from RR‐MS patients, compared to non‐treated MS patients and healthy controls, as well as compared to patients treated with two other first‐line drugs for RRMS (i.e., IFN‐β and teriflunomide).^69^ However, the number of endothelial, platelet, and monocyte plasma‐derived EVs increased in MS patients treated with IFN‐β or natalizumab.^70^ It is possible that these differences are due to the different methods used to isolate EVs.

Lastly, in serum‐derived EVs, Ebrahimkhani et al. identified two miRNA signatures, which permitted RR‐MS to be distinguished from progressive MS, showing that EVs are informative biomarkers in identifying MS subtypes.^71^ Similarly, miRNAs profiling could also be used to monitor response to IFN‐β therapy, since treated RR‐MS patients showed up‐regulation in the serum of 2 miRNAs (e.g., miR‐22‐3p and miR‐660‐5p), compared to the untreated group.^72^

Despite the issues addressed above, in connection with the use of EVs as biomarker for MS, correlation of their levels with clinical outcomes (i.e., magnetic resonance) is lacking, and it is only documented in a few studies. Since the efficacy of response to therapy relies on the EV count, standardization of the methodology is also required to obtain comparable results.

### 3.3 EVs for MS therapy

Administration of EVs purified from human adipose‐derived MSCs induced repair pathways in CNS and promoted recovery in Theiler's murine encephalomyelitis virus (TMEV)‐induced demyelinating disease, which is the mouse model of progressive MS.^73^ Moreover, it has recently been shown that intravenous injection of MSC‐derived exosomes ameliorated an experimental rat autoimmune encephalomyelitis (EAE) model by decreasing T helper (Th)1 and Th17 cytokine levels, and inducing polarization of microglia towards an anti‐inflammatory phenotype.^74^ In vitro, MSC‐derived MVs induced the secretion of tolerogenic cytokines, such as IL‐10 and TGF‐β, and inhibited T cell proliferation in EAE splenocytes.^75^

In a prophylactic setting, administration of EVs isolated from adipose stem cells ameliorated chronic EAE without inhibiting T cell proliferation, but blocked T cell trafficking to the CNS.^76^

Other sources of therapeutic exosome are those released by stimulated dendritic cells (DCs). Nasal administration of low‐dose exosomes derived from in vitro IFN‐γ‐stimulated DC sustained remyelination. This important achievement was mediated by miRNA‐219, impacting on oligodendrocyte differentiation and inducing increased myelin production.^77^ Interestingly, the same group demonstrated that serum‐derived exosomes of young mice increased oligodendrocyte proliferation when applied onto hippocampus slice cultures in vitro, and enhanced re‐myelination when injected nasally in elderly rats. Characterization of the cargo of those exosomes identified 17 miRNAs, interestingly including miRNA‐219.^78^ These interesting findings are a milestone in the field, and shed new light on the use of exosomes as a *“*fountain of youth*”* for oligodendrocytes in chronic and aged‐MS. As stated above, EVs can also act as a delivery vehicle to carry anti‐inflammatory molecules/drugs. Intracisternal injection of engineered microglia‐derived EVs containing IL‐4, and overexpressing a protein increasing their uptake by myeloid cells and astrocytes, resulted in the amelioration of EAE.^79^ Zhuang et al. showed that intranasal administration of curcumin encapsulated in exosomes effectively reached the target and reduced the severity of EAE.^80^

## 4 RHEUMATOID ARTHRITIS

RA is a chronic autoimmune disease, characterized by synovitis and joint damage, leading to loss of function and increased morbidity and mortality.^81^, ^82^ It is the result of a complex dysregulation of the immune system that involves both innate and adaptive immunity, characterized by chronic inflammation and development of autoimmunity.^83^

In recent decades, improved knowledge of the mechanisms underlying the pathogenesis of RA has enabled potential targets for disease treatment to be identified, leading to the development of novel target therapies. These have had a groundbreaking effect on RA's natural history. However, the pathogenesis is still poorly known, and predictive models enabling treatment strategy to be tailored on an individual basis are lacking.

### 4.1 EVs in RA pathogenesis

In recent years, a variety of evidence has pointed towards a potential pathogenetic role of EVs in RA. Firstly, EVs are a potential source of autoantigens (Fig. 2C), which is particularly relevant in the specific setting of RA, since this disease is characterized by the development of typical autoantibodies, including anti‐citrullinated protein (ACPA) and anti‐rheumatoid factor antibodies.^84^ Citrullination is a post‐translational modification of arginine mediated by the activity of the enzyme peptidylarginine deiminase (PAD). Interestingly, EVs isolated from synovial fluid (SF) of RA patients are carriers of citrullinated peptides,^85^ including PAD‐2 and PAD‐4, and are expressed in synovial fluid proportionately to the degree of inflammation.^86^ More recently, EVs from the SF have been characterized, demonstrating that they differ in size from other EVs, being larger; they are also carriers of immune complexes. Interestingly, the more abundant EVs in the SF express CD41, probably being derived from platelets, and they promote migration, invasion, and adhesion to extracellular matrix (ECM) of RA fibroblast‐like synoviocytes (FLS).^87^

Platelet‐derived EVs are not only sources of autoantigens, but also exert a pro‐inflammatory effect.

Boilard et al. demonstrated that platelet‐derived EVs enhance the production of IL‐6 and of the neutrophil chemoattractant IL‐8 by FLS. These two factors play a relevant role in maintaining joint inflammation; the effect of EVs on FLS is mediated by IL‐1.^88^ Moreover, EVs isolated from TNF‐α‐treated T cells and monocytes have been shown to stimulate FLS production of cyclooxygenase 2 (COX‐2), microsomal prostaglandin E synthase 1 (mPGES‐1), and prostaglandin E2 (PGE2).^89^ This effect is the combined result of activation of pro‐inflammatory intracellular pathways (i.e., NF‐κB and JNK) and of the increased availability of substrate for COX‐2 activity, since EVs are able to transport arachidonic acid from leukocytes to FLS.^89^ TNFα‐expressing EVs are characterized by a cytotoxic effect on endothelial cells, possibly contributing to the accelerated atherosclerosis which is a typical extra‐articular feature of RA.^90^

A further pathogenetic element in RA is neoangiogenesis; the development of new blood vessels contributes to pannus development and represents a typical feature of inflamed synovial tissue*. In vitro*, EVs isolated from Jurkat cells and U937 cells induce the expression of pro‐angiogenic chemokines (C‐X‐C motif (CXCL1, CXCL2, CXCL3, CXCL5, and CXCL6) in RA synovial fibroblasts (RASFs). The study authors also demonstrated that in an in vivo bio‐chamber assay, supernatants from RASFs co‐cultured with EVs stimulated angiogenesis.^91^ EVs produced under inflammatory conditions by leukocytes are able to upregulate the expression of matrix metalloproteases (MMPs), which are involved in matrix degradation.^92^ EV‐associated MMPs might alter the EV cargo by ectodomain shedding, which is a mechanism whereby membrane‐anchored proteins are released from the cell surface as soluble proteins, usually decoy molecules, or by exerting proteolytic activity following uptake by target cells, or directly contributing to the degradation of extracellular matrix proteins surrounding cells.^93^

Lastly, growing evidence links miRNAs to the pathophysiology of RA, particularly miR‐155 and mi‐R146a.^94^ DC‐derived EVs carrying these two miRNAs can be taken up by recipient immune cells, thus regulating the inflammatory response in the recipient cell: indeed, miR‐146a inhibits, while miR‐155 promotes, endotoxin‐induced inflammation in mice.^95^ Interestingly, the same miRNAs, along with miR‐323a‐5p, and miR‐1307‐3p, have been reported up‐regulated in RASF‐derived exosomes upon TNF DC‐ stimulation.^96^

More recently, mi‐R17 has been claimed as a potential pathogenetic effector in RA, since it is more abundant in RA‐EVs than in those of healthy controls, and suppresses Treg induction by inhibiting the expression of TGF‐β II (TGFBR II) possibly contributing to the impairment of Treg homeostasis in RA patients.^97^

### 4.2 EVs and RA biomarkers

EVs have been postulated as potential biomarkers in RA: both the total plasma concentration of EVs and the concentration of specific subsets of EVs have been used in the past. In the latter case, some studies have analyzed the potential role of specific subsets deriving from a defined cell source, while others have considered the amount of EVs carrying specific target molecules.^98^

Looking more deeply at the current literature, the first report about EV levels in RA occurred in 2002, when Knijff‐Dutmer et al. reported that, in a cross‐sectional group of 19 RA patients, plasma platelet‐derived microparticles (PMP) were significantly higher than in healthy controls (HC). Moreover, PMPs display a correlation with disease activity score (DAS), since their amount increases in patients with active versus inactive RA.^99^, ^100^ In the same year, a Japanese group confirmed that RA patients show increased levels of plasma PMPs, which decrease after leukocytapheresis (LCAP), paralleling a disease activity improvement. Interestingly LCAP was associated with an increase in the circulating levels of granulocyte‐derived EVs, which are thought to play an immune‐regulatory role.^101^, ^102^ However, these findings have not been replicated in other reports: in 2015 Rodriguez‐Carrio reported increased total plasma EVs in RA patients with respect to HC, but this increase was related to a significantly larger amount of endothelial and granulocyte‐derived EVs, rather than to PMP which conversely were comparable between groups.^103^ Furthermore, T‐cell‐derived EVs have been reported increased in RA.^104^, ^105^

Taken together, these findings show that RA patients have increased levels of circulating EVs, although evidence is conflicting concerning the source from which these EVs are released.

A possible explanation of these discrepancies might lie in the different features of RA patients enrolled in the studies: the profile of the different subsets of EVs varies with disease activity. In RA patients with a high DAS28 (> 5.1), the plasma concentration of monocyte, platelet, endothelial cell, and B lymphocyte‐derived EVs has been shown to be significantly higher than in healthy controls. Patients with moderate disease activity (DAS28 3.2‐5.1) showed enhanced production of plasma monocyte and endothelium‐derived EVs, whereas there was no difference versus healthy controls when DAS28 fell in the range of low disease activity. Consistently, the study authors reported a direct association between all these subsets of EVs and DAS28.^106^

Another study showed that, in seropositive patients for Rheumatoid Factor (RM), immunoglobulin (Ig)M RF may be detected on plasma EVs. This subset of seropositive RA patients is characterized by higher disease activity and systemic inflammation compared with seronegative patients, but also compared with seropositive patients without RF^+^ EVs. This suggests that EVs may serve as prognostic biomarkers in this context.^107^ Consistently, seropositive RA patients have higher proportions of immune complexes (IgG^+^IgM, IgG^–^IgM^+^, and IgG^+^IgM^+^) and citrullinated proteins on the surface of their EVs, deriving from both platelets and leukocytes.^108^ Finally, an increased concentration of plasma EVs exposing complement activation particles, such as C1q, or activator molecules such as reactive C protein (CRP) or serum amyloid‐P (SAP), have also been reported to be increased in RA, remaining unchanged despite intensive anti‐rheumatic treatment.^109^

A last potential application of EVs, as already shown for MS, is the prediction of patient response to a specific treatment. A growing number of biological disease‐modifying anti‐rheumatic drugs (bDMARDs) and targeted synthetic DMARDs (tsDMARDs) for RA, have been licensed during 2019, becoming part of everyday clinical practice for the rheumatologst^110^; this is important because all patients do not respond equally to a specific therapy.

Nowadays, it is a common perception that RA is not the same in all subjects, and that some patients are non‐responsive to the blockade of a specific pathway, whereas they are sensitive to other therapeutic strategies. No prognostic tools are available to predict whether a specific patient will respond or not to a specific treatment. A significant line of current research in the field of rheumatology aims to define predictive models to help the clinician to allocate the right treatment to the right patient. Different options have been proposed, essentially related to a pathobiology‐driven strategy, based upon either a synovial biopsy,^111^ or a liquid biopsy strategy, relying on the identification of circulating biomarkers. Specifically, in terms of EVs, there is evidence that some miRNAs might be useful for this purpose.^112^ Evidence suggests that EV‐derived miRNAs could be used as potential prediction of response to therapy in RA patients. Methotrexate treatment (DMARDs) can increase the whole blood expression of some miRNA^113^: hsa‐miR‐132‐3p, hsa‐miR‐146a‐5p, and hsa‐miR‐155‐5p have all been proposed as potential predictors of response,^114^ including in patients treated with anti‐TNF/cDMARD. However, these data are very recent and validation on larger cohorts will be required.

Interestingly, a specific plasma miRNA signature (miR‐23 and miR‐223) has been identified that may serve as predictor and biomarker of response to anti‐TNFα/DMARDs combination therapy.^115^ Specific miRNAs have also been identified in RA patients as predictors of response to adalimumab or to etanercept, which are both monoclonal antibodies that inhibit TNF‐α.^116^ Conversely, Krintel et al. identified the combination of low whole blood expression of miR‐22 and high expression of miR‐886.3p as a predictor of good response to EULAR.^117^

Lastly, in a further study, an elevated level of plasma miR‐27a‐3p before treatment was significantly associated with remission at 12 months, in a group of patients treated with adalimumab and methotrexate.^118^

It is evident that these findings are contradictory and that, although promising, the use of the EV signature for treatment allocation is still far from being clinically applicable. However, the preliminary results obtained on discovery cohorts deserve further validation in the near future, to better understand the potential usefulness of EV characterization in clinical practice.

### 4.3 EVs for RA therapy

In the context of autoimmune disorders, RA is a perfect model for targeted drug delivery: there are drugs targeting known molecules at the joint level, whose effectiveness and safety might bring enormous benefit upon direct delivery to the joint. Moreover, some EVs might have a beneficial effect on autoimmune disease, related to the anti‐inflammatory effect of the miRNAs expressed by the microparticles per se, or by regulatory proteins carried by the EVs.^119^

The first report dealing with the topic of EVs implication in RA treatment dates to 2009, when Bianco *et al*. reported the positive effect on collagen‐induced arthritis (CIA) of EVs deriving from “tolerogenic” DC, treated with IL‐10.^120^ With a similar approach, the same group later reported the beneficial effect on CIA of DC‐EVs transfected with IL‐4.^121^ Lastly, they also published a study in which DCs were adenovirally transfected with CTLA‐IgG or indoleamine 2,3‐dioxygenase (IDO), a tryptophan degrading enzyme important for immune regulation and tolerance maintenance. The exosomes produced by DC were isolated and administered to CIA mice, with a significant improvement of the inflammatory joint disease.^122^

It has been shown that neutrophil‐derived EVs are abundant in the SF of RA, and that they overexpress the proresolving, anti‐inflammatory protein annexin A1 (AnxA1); the addition of these EVs to chondrocyte cultures in vitro led to the activation of anabolic pathways and cartilage protection. In a murine model of cartilage damage, this was reflected by the reduced cartilage degradation observed in mice receiving intra‐articular injection of AnxA1^+^ EVs.^101^ Although cartilage and bone destruction are irreversible processes, since cartilage repair capacity is poor, EVs deriving from MSCs are a particularly promising therapeutic tool in joint repair, since they have been shown to maintain some of the regenerative properties characterizing their cells of origin.^123^ Thus, intra‐articular administration of MSCs EVs led to an improvement, in a murine model of osteochondral defect.^124^

The therapeutic effect of EVs might in some cases belong to the miRNA expressed within the EV. In 2015, the beneficial effect of bovine‐milk‐derived EVs (BMEVs) on a murine model of spontaneous arthritis in IL‐1Ra deficient mice, and in CIA, was first observed. These BMEVs, expressing immunoregulatory microRNA (miR‐30a, ‐223, ‐92a), delayed the onset of arthritis and diminished cartilage pathology and bone marrow inflammation in both models.^125^ Moreover, it was reported that EVs carrying miRNA‐150‐5p have a potential therapeutic effect on RA because they are able to lower the expression of VEGF and MMP14 and, consistently, inhibit angiogenesis and FLS migration in an in vitro assay. In vivo, the administration of miRNA‐150‐5p EVs is able to improve the clinical phenotype of collagen‐induced arthritis in a murine model.^126^

Lastly, EVs can be used as liposomes, to deliver a specific drug directly to the joint. In 2014, a synovium‐specific targeted liposomal drug loaded with glucocorticoids was employed to specifically target FLS and endothelial cells. This strategy was effective in vivo, suppressing the inflammatory response in affected joints,^127^ and confirming that EVs are a promising vehicle for drug delivery in RA.

## 5 SYSTEMIC LUPUS ERYTHEMATOSUS

SLE is a chronic AD characterized by the production of antinuclear antibodies (ANAs), anti‐double‐stranded DNA antibodies, but also autoantibodies targeting cytoplasmic antigens. SLE is a multisystemic inflammatory disease, affecting several organs, in particular the kidneys. From the clinical standpoint, SLE may present unpredictable outcomes: periods of remission and flares alternate over time, and a mild involvement limited to the joints may be followed by severe and widespread organ damage.^128^ SLE diagnosis is achieved through clinical observations and laboratory examinations, and therapy is determined depending on disease manifestations and severity. Clinical prognosis and treatments have improved over time, and treatment chiefly comprises the use of steroidal and nonsteroidal anti‐inflammatory drugs, immunosuppressive agents, and biologic agents.^129^ However, clinicians still lack biomarkers for prediction of disease outcome, and several studies are aimed at addressing this issue.

### 5.1 EVs in SLE pathogenesis

Anti‐ANAs and anti‐DNA antibodies are associated with the severity of SLE.^130^ ANAs can bind to DNA, RNA, proteins in the nucleus, and form immune complexes (ICs) of nucleic acids. ICs constitute the serological hallmark of SLE and can drive its pathogenesis by depositing within target tissues, in particular the kidney.^130^ ICs settle in the tissues, where they either to fix complement or induce cytokine production (most prominently type 1 interferon), thus inciting inflammation. Nucleic acids usually associate to proteins both inside and outside the cell.^131^ Thus, the loss of B cell tolerance to DNA and/or chromatin represents a major mechanism of SLE pathogenesis. Moreover, anti‐DNA antibodies can also cross‐react with other self‐antigens^132^ (Fig. 2D). EVs are thought to represent the different sources of autoantigens are involved in the formation of ICs in SLE^133^: nuclear antigens have been found as components of EVs cargo, and this can provide a new perspective to clarify how they form ICs and drive SLE pathogenesis.^131^ Dysregulated cell death and defective clearance of dying cells have been proposed as contributing to autoantigen generation and induction of autoantibodies. In addition, this could be associated with the development of lupus glomerulonephritis (LN), which is one of the most common and severe complications in SLE.

DNA from apoptotic cells is degraded by the intracellular deoxyribonuclease (DNASE): it has been observed that the deletion of the variant 2 of this enzyme (DNASE2) in mice causes IFN‐driven autoinflammation.^134^ Conversely, DNASE1L3‐deficient mice rapidly develop autoantibodies both to DNA and to chromatin, followed by an SLE‐like disease. Several studies have indicated that genomic DNA of apoptotic cells is incorporated into plasma EVs.^135^, ^136^, ^137^ These vesicles expose chromatin on their surface and therefore may represent antigens for circulating DNA‐reactive B cells.^138^

### 5.2 EVs and SLE biomarkers

Autoimmunity is characterized by cell activation, and cell stimulation leads to the shedding of phosphatidylserine (PS)‐rich EVs, which may be suitable biomarkers for SLE and other ADs. PS may thus be used to detect EVs in the body fluids.^139^ In SLE, autoantibodies to chromatin, including nucleosomes, usually serve as especially sensitive biomarkers of the disease.^140^ Recently, using the proteomics approach, Ostergaard and colleagues showed that the cargo of plasma circulating EVs in SLE patients contained an increase of specific proteins (e.g., complement, IgG, microtubule proteins, fibronectin, and desmosomal) compared to healthy controls, and these expression patterns correlate with the disease progression.^141^ Circulating EVs were detected in the plasma of SLE patients using multiparametric flow cytometry. These studies have outlined novel subpopulations of platelet, endothelial, and leukocyte‐derived EVs, some of which have clinical and serological correlations. In particular, frequencies of platelet‐derived EVs (characterized as CD41^+^, and CD41^+^‐CD40L^+^), were found to be increased compared with healthy controls, regardless of disease activity.^108^ Furthermore, endothelial activation and damage is commonly observed in SLE patients, and is related to the development of nephropathies and vascular diseases. In this connection, it has been found that endothelial‐EVs (detected as CD144^+^, VCAM^+^ ) and tissue leukocyte‐EVs (TF^+^‐CD45^+^) are highly up‐regulated compared to healthy controls, and these markers directly correlate with the degree of inflammation, but also glomerulonephritis and vascular dysfunction.^141^ In addition, they have been proposed as markers of cytoskeletal composition defects.^141^

The protein signature of endothelial‐EVs could also be used as biomarker of the activity and progression of SLE, and of the presence of possible complications. As mentioned above, one of the most affected organs in SLE is the kidney: the possibility to assess glomerular damage by screening circulating biomarkers, instead of invasive biopsy, would offer a great advantage in monitoring disease progression. Urine is the ideal biological fluid for detecting new biomarkers, thanks to its noninvasive collection. Several studies have shown that the cargo of urine EVs is enriched with miRNA, which can be used as potential biomarkers. Perez‐Hernandez et al. reported that urine miR‐146a was markedly increased in SLE patients, and could be used to discriminate patients with active lupus nephritis from those without.^142^ Solè et al. investigated the expression level of miR‐29c, and found a negative correlation between urinary EV miR‐29c level and histological chronicity index and glomerular sclerosis, but not renal function. They proposed that urinary exosomal miR‐29c might be used as a non‐invasive biomarker of early progression to renal fibrosis.^143^ In other studies, increased expression levels of miR‐26a and miR‐29c in human glomeruli or urine were found as biomarker of podocyte injury and LN, respectively.^144^, ^145^ Conversely, miR‐146a down‐regulation in the plasma and serum from SLE patients can be associated with alterations in the type 1 IFN pathway, and maybe a marker of disease progression.^146^ However, other parallel studies report that miR‐146a in urinary EVs is overexpressed in the glomeruli of LN patients.

More recently, mRNA for CD2AP (CD2 Associated Protein), a protein that regulates the actin cytoskeleton, which is involved in the glomerular filtration barrier, was shown to be down‐regulated in SLE patients and correlated with proteinuria.^141^ Induction of podocyte damage in rats produced an increase of cystatin C mRNA levels in EVs, which is a marker of glomerular damage. Lastly, several miRNAs have been reported to participate in the regulation of DNA methylation, which plays a pathogenetic role in SLE. Interestingly, EVs from T cells delivering miR‐148a, miR‐126, miR‐29 b, and miR‐21 can inhibit DNA methyltransferase 1. Lastly, in the light of the known involvement of notch signaling in SLE pathogenesis, a study has reported that patients with glomerular kidney diseases have elevated levels podocytes‐EVs carrying ADAM10, which is a metalloproteinase involved in the Notch signaling complex.^147^

### 5.3 EVs for SLE therapy

Over recent years, EVs derived from MSCs have emerged as cell‐free therapeutic agents to treat autoimmune and inflammatory diseases. Thanks to their immunomodulatory properties, MSCs have been widely used in clinical trials as immunosuppressive agents. As recently demonstrated, MSCs exert their immunomodulation function through EVs, making them a promising tool for AD therapy in several human diseases. However, clinical results have provided evidence of potential side effects, including neoplastic transformation, following MSC therapy. In this context, since EVs are less immunogenic than their parent cells, they might be a valid alternative. In this connection, several pre‐clinical studies in animal models of AD have exploited the therapeutic potential of MSC‐EVs. The results show that they are effective in improving therapeutic effects and decreasing the side effects Thus the use of novel targeted immunotherapies, based on MSC‐EV transfer of RNA and protein for immunosuppression, is highly attractive. They may act as immunologically active agents by releasing anti‐inflammatory cytokines, and also by modulating Toll‐like receptor signaling.

Regarding SLE, many preclinical studies have assessed MSC transplantation in murine models. In particular, in a Fas‐deficient MRL/lpr mouse model of SLE, Liu et al. employed MSC‐EVs to transfer the Fas receptor to the bone marrow; thus, by reducing intracellular miR‐29b levels, they ameliorated osteopenia.^148^

Furthermore, in mice with acute kidney injury, treatment with MSC‐EVs resulted in the prevention of chronic tubular inflammation and of renal damage, and also prolonged survival.^149^

Intriguingly, the therapeutic potential of MSC‐EVs has been tested in a human clinical trial: a cohort of patients with chronic kidney disease were recruited and subjected to the administration of autologous MSC‐EVs. Analyzing urinary parameters (i.e. blood urea, serum creatinine, urinary albumin creatinine ratio, and estimated glomerular filtration rate) they monitored kidney functions. Lastly, to evaluate the treatment‐induced improvement of inflammatory immune activity, they evaluated the following parameters: TNF‐α, TGF‐β1, and IL‐10, which are all involved in immune‐regulation and inflammation. Interestingly they discovered that treated MSC‐EVs patients exhibited significant increases in plasma levels of TGF‐β1, whereas TNF‐α was significantly decreased, suggesting amelioration of the inflammatory immune reaction.^150^

## 6 TECHNICAL ISSUES

EVs have attracted great interest as important contributors to the autoimmune response, not only at its onset, but also for their immunomodulatory activity, and their potential as biomarkers for disease activity or response to therapy. However, data from published studies are very variable, being biased by the difficulties involved in EV isolation and characterization.

It is still challenging to isolate circulating EVs with good recovery and without contamination from proteins and lipoproteins. To date, most isolation protocols are based on differential centrifugation. However, high velocities generate protein and vesicle aggregates. Further, isolation of vesicles from plasma or serum by density‐gradient ultracentrifugation results in co‐isolation of high‐density lipoprotein (HDL), and isolation of HDL results in co‐isolation of vesicles. There is thus an urgent need for a simple and fast protocol to isolate vesicles from human samples. Determining the optimal strategy for isolating EVs is a critical step toward retrieving the maximal amount, while ensuring the recovery of all different vesicular subtypes and subpopulations, including the rare ones.^151^

According to the recent MISEV2018 guidelines,^152^ several methods have been proposed for the detection, quantification, and characterization of EVs. These include differential ultracentrifugation, size‐exclusion chromatography, immunoaffinity capture, microfluidics, and the use of exosome commercial kits. However, different methods of isolation might lead to different outcomes or discordant results, even starting from the same source. Another important issue to be addressed is that, during manipulation of biological samples (i.e. from blood to serum), platelets and other cells may release EVs owing to their damage, activation, or disruption. As a consequence, direct evaluation on the blood would be preferable. In this connection, flow cytometry is one of the most promising methods. Indeed, this technology can be used to characterize EVs in liquids, including blood, plasma, and other biologic fluids, as well as in solid tissues. One of the main advantages of cytometry is the possibility to detect rare populations and distinguish them from abundant ones. Moreover, the technology enables the populations to be individually sorted, and they can then be typed in depth through ‘omic’ analyses (proteomics, transcriptomics, metabolomics). Interestingly, very recent studies described a new method for the identification and sorting of EVs by flow cytometry, using a lipophilic cationic dye that diffuses through plasma membranes and directly binds to EVs (Pan‐EV dye) in unmanipulated body fluids (tears and CFS) in combination with specific EVs surface markers.^65^, ^153^, ^154^

Further experiments will be needed to validate this method in the AD setting, and compare the results with those obtained using conventional procedures accepted by the scientific community.

## 7 CONCLUDING REMARKS

Currently, there are still significant gaps in our knowledge of the mechanisms underlying the pathogenesis of autoimmune diseases, in particular during the prodromal phases, and few initiating autoantigens have been identified. Indeed, ADs are usually only diagnosed sometime after epitope spreading, a process during which the immune response, begun by the autoantigen, is taken over by new T or B cell specificities. This makes it difficult to identify the “initiating antigen”. Such identification would be of great utility, since preclinical studies have revealed a window of therapeutic opportunity before the overflow of epitope spreading. Identifying the starting antigen would help to fully understand the etiology and the pathogenesis of autoimmune diseases, and to develop better immunotherapeutic approaches. In the opinion of the reviewers, a great revolution in the search for AD initiating autoantigens will come from studying rare subpopulations of EVs. Alongside the mechanisms that have been described for individual diseases (T1D, MS, RA), an initiating role may be ascribed to molecular mimicry, in which a foreign antigen (usually a viral antigen) shares sequence or structural similarities with self‐antigens. This concept may be extended to all foreign molecules. Interestingly, EVs are highly conserved during evolution, and are produced by organisms of all kingdoms, i.e., bacteria, virus, plant, and animal cells. The nutritional value of foods depends on the composition of the gut microbiome, but dietary components, in turn, shape the composition and functional status of the microbial community. It is thus possible, and has partially been proved, that not only damaged tissues but also microbiota and foodstuffs from the gut lumen deliver EVs or their contents to epithelial cells, and/or enter the systemic circulation, whence they may be delivered to different proximal or distant organs, eliciting a variety of immunological and metabolic responses^155^(Fig. 1). Therefore, the important role of gut microbiota in ADs is still a subject of intense investigation, in order to improve the knowledge of the complex network of active molecules secreted by intestinal bacteria and their impact on gut epithelial cells, GALT, and distant organs.

## AUTHORSHIP

All the authors wrote and approved the final manuscript.

## DISCLOSURES

The authors declare no conflict of interest.
